# Examining the Impact of E-Government on Corporate Social Responsibility Performance: The Mediating Effect of Mandatory Corporate Social Responsibility Policy, Corruption, and Information and Communication Technologies Development During the COVID era

**DOI:** 10.3389/fpsyg.2021.737100

**Published:** 2021-10-12

**Authors:** Andrianarivo Andriandafiarisoa Ralison Ny Avotra, Ye Chengang, Tsimisaraka Raymondo Sandra Marcelline, Ali Asad, Yang Yingfei

**Affiliations:** ^1^Business School, Zhejiang Wanli University, Ningbo, China; ^2^Business School, University of International Business and Economics, Beijing, China; ^3^School of Management, Shenzhen University, Shenzhen, China; ^4^Ningbo University of Finance & Economics, Ningbo, China

**Keywords:** mandatory CSR policy, corruption, ICT development, e-government on CSR performance, Covid era

## Abstract

During the Covid-19 era, this research will explore and analyze the link between e-government and corporate social responsibility. In addition, mandatory corporate social responsibility, institutional quality, information and communication technology, and corruption as mediators will also be explored in this study. This research seeks to answer the issue of how e-government affects corporate social responsibility and how other mediating variables might influence this connection. Furthermore, this study developed a total of 13 hypotheses based on these questions, 4 of which have mediating effects. The theoretical underpinning for the proposed study paradigm is provided by stakeholder theory, which has been established based on prior literature. The general philosophy is positivism, and the research has a deductive nature. The information was gathered from 305 managers across four industries: information technology, online services, online education, and logistics and supply chain. Data was collected using a random convenience sampling approach. The Partial Least Square Sequential Equation Modeling (PLS-SEM) research analysis approach was applied in this study for the analysis. The measurement step demonstrated that all constructs and indicators are valid and trustworthy enough to be utilized in the future. The results of the structural model evaluation revealed that e-government had a negative influence on corporate social responsibility, with three of the four mediating roles proving to be completely mediated. As a result, the government and relevant stakeholders should take these results into account when formulating e-government policies.

## Introduction

E-government has emerged as the presiding medium for connecting, managing, and servicing citizens (Bwalya and Healy, 2010). According to the World Bank, e-government is the use of Information and Communication Technologies (ICTs) to improve the business operations and service provision of government agencies. The growth of e-government depends on various factors such as information technology, human resource management, legislative willingness, infrastructure, and public trust (Khalil, 2011). E-government promotes citizens’ participation in public administration, enhances awareness of citizens toward government programs, improves the transparency of public decisions, and reduces corruption (Shim and Eom, 2008; Sabani et al., 2019). As a consequence, governments around the world have been working hard to adopt specific e-government initiatives for e-government development (Deng et al., 2018).

Conversely, corruption has become a global issue, posing a threat to transparency, governance, and accountability (Wellalage et al., 2019). The literature on e-government provides evidence on the importance of e-government in combating corruption and implying that government digitalization may help to fight against corruption (Nam, 2018; Linhartová, 2019; Adam, 2020). Zhang and Sun (2013) argued e-government can also be utilized to enhance public administration’s openness and legitimacy as well as to combat all types of corruption. In this regard, government services can improve government operational efficiency by decreasing its corruption levels (Suhendi et al., 2020). This demonstrates that e-government has a dominant part in anti-corruption policies and helps organizations to enhance their corporate social responsibility (CSR) performance (An et al., 2021). In this era, ICTs are becoming progressively popular. The rapid growth of ICT may provoke both open opportunities and challenges in terms of creating, accessing, processing, and utilizing the correct information. It has been argued that ICTs play a significant role in eliminating corruption due to the vast range of digital options available worldwide (Alhammadi, 2009; Park and Kim, 2020). ICT’s development is also found as a crucial component of a country that may serve as a foundation for e-government implementation. As ICTs development creates an effective mechanism for ICT access and structuring e-government (Huo et al., 2021; Xialong et al., 2021). Therefore, e-governments should employ effective information technologies for their better performance.

Government and companies are being widely involved in attempts to resolve environmental and social concerns and reduce corporate vulnerabilities. CSR performance may help to reinforce, normalize, and eliminate economic disparities in a society (Bapuji et al., 2020). The corporations that paid more attention to the development of CSR activities enjoy a good reputation among their stakeholders (Lin-Hi and Blumberg, 2018). Particularly in this era of ICTs development, any information related to corporations like their opinions on CSR can influence a stakeholder’s decisions (Elfeky, 2017). While arguments related to mandatory CSR policy are continuously shifting in response to academic research agendas, corporate scandals, and economic climate (Chen et al., 2021; Li et al., 2021; Wu et al., 2021).

In the prior literature, the association between corruption and e-government has been explored extensively, and concludes as e-government initiatives do not eliminate all forms of corruption (Ali et al., 2021; Maqsoom et al., 2021b). While limited studies have documented the influence of ICT development and e-government on corruption (Srivastava and Vyas, 2015; Srivastava et al., 2016). It has been stated that governments have eradicated and reduced corruption levels significantly as a result of e-government development platforms (Ojha and Palvia, 2012). More corruption may be reduced via initiatives that encourage transparency and accountability (Mistry, 2012). This shows that the literature has not addressed the influence of e-government development on CSR performance through the mediating role of ICTs, CSR policy, corruption, and institutional quality. In order to address this gap, this study develops a model to empirically evaluate the influence of e-government development on CSR performance through the mediating role of considered variables.

The remainder of the paper is organized as follows: section “Review of Literature” discusses the literature review on considered variables and the development of the hypothesis. The section “Research Methods” is related to research methodology, which is employed to test the hypothesis, and the section “Data Analysis” discusses the study analysis and results. The last section brings us to the Discussion and Conclusion.

## Review of Literature

### E-Government Development

E-government refers to the government’s use of ICTs to improve citizen access to and delivery of government activities and services (Bélanger and Carter, 2012). E-government also refers to the use of technology to boost public services and communication, as well as making government more effective and efficient (Krishnan et al., 2013). More it refers to a broad variety of government functions and activities that are affected by the ongoing integration of ICTs with certain other management paradigms (Ziemba et al., 2016). The usage of e-government aids the anti-corruption fight against self-serving tax dodging by state officials and others who plot large political corruption schemes (Singh et al., 2010). E-government can be considered from multiple perspectives such as e-society, e-administration, and e-citizens. Its successful implementation can lead to increased internal efficiency, stakeholder satisfaction, and service improvement because of standardized operational processes, the transformation of paper-based information into electronic form, and divergent databases (Nawaz et al., 2019; Hao et al., 2020b; Wu et al., 2020). Such types of activities help the stakeholders in easy access to government services. More in e-government platforms public services are not only supplied by the government but also incorporate citizens’ participation enabled by ICTs development (Anttiroiko et al., 2014).

### Mandatory Corporate Social Responsibility Policy

Corporate social responsibility is described as a voluntary commitment by corporations to contribute to a better society and cleaner environment. Several governments have lost patience with a firm’s willingness to make adequate voluntary contributions to CSR activities; therefore, governments have taken steps to make mandatory CSR policy for promoting transparency and accountability. Gatto (2002) claims that mandatory CSR enhances corporate commitment and empowers numerous external stakeholders around firms. The use of mandatory CSR policy has been recommended as a way to persuade certain businesses to invest in CSR initiatives (Mukherjee et al., 2018). Scholars have argued that mandatory CSR is based on a country’s particular hard law rather than a soft law based on self-regulation (Scherer et al., 2016). Corporations are willing to turn the expectations of nation-states in terms of socioeconomic growth. The ramifications of mandatory CSR policy allow company executives to better understand and handle the concerns of local stakeholders and their intermediaries (Ioannou and Serafeim, 2012).

### Corruption

Corruption is characterized as the use of state authority or position for personal gain (Rose-Ackerman, 1999). Whereas Rodriguez et al. (2006) termed corruption as misuse of economic and social power for personal objectives. The corruption may be caused by a monopoly of power, lack of accountability, discretion, and transparency (Kaufmann, 2011). Hence, the companies that deliberately operate in illegal and corrupt circumstances endanger their reputations (Ioannou and Serafeim, 2012; Keig et al., 2015). Corruption at the organizational level is usually performed by the employees and managers on behalf of the corporation (Hao et al., 2020a; Nawaz et al., 2020a). Managers and employees are more likely to participate in unlawful conduct when the company’s transparency and morale are low. The literature on e-government (Nam, 2018; Linhartová, 2019; Adam, 2020) provides evidence on the importance of e-government in combating corruption and suggesting that government digitalization may help to fight against corruption.

### Information and Communication Technologies Development

Information and Communication Technologies are becoming increasingly popular due to the development of e-government. ICT as a technical solution can significantly enhance the effectiveness of individuals and organizations (Matmati, 2001). Indeed, ICT has an influence on working habits and practices as well as relationships within an organization. Moreover, ICTs can also be used as a measure of government digitization, more digital operations, procedures, services, transactions, and applications lead to a higher level of e-government (Uyar et al., 2021). The ICT infrastructure comprises basic ICT access, which includes personal computers, telephone lines, Internet penetration and access in remote regions as well as the speed at which the public may access the Internet. ICTs development is also found as a crucial component of a country that may serve as a foundation for e-government implementation. As ICTs development creates an effective mechanism for ICT access and structuring e-government (Nawaz et al., 2021).

### Institutional Quality

The institutional quality of a country serves as an intermediary for efficient e-government development (Adam, 2020). ICTs in the context of e-government may be a cost-effective and easy way of ensuring open and transparent government which leads to reduced corruption in systems (Bertot et al., 2010). Countries that have achieved success in enacting transparency legislations have been linked to based efforts via e-government (Keig et al., 2015). ICTs promote good governance, boost reform-oriented efforts, improve connection among government personnel as well as monitor and manage government employee and project behavior. As a result of e-government advancement, institutions can become more transparent in their managing information and procedures. A strong country requires adequate financial market laws, a robust rule of law, intellectual property rights, and high-quality institutions capable of successfully combatting corruption as compared to weak institutions (Liu et al., 2021;Zheng et al., 2021).

### Corporate Social Responsibility Performance

Corporate social responsibility is a set of comprehensive policies related to corporate ethics, community service, and the environment that are integrated into a company’s operations (Carroll, 1970). By implementing CSR activities efficiently firms can gain multiple benefits such as long-term self-interest, greater stakeholder’s interest, and enhanced public image (Jain, 2020). Corruption at the organizational level is usually performed by the employees and managers on behalf of the corporation (Luo, 2005). Managers and employees are more prone to participate in unlawful conduct when the company’s transparency and morale are low. Corruption and corporate activities are endogenously influenced by economic conditions and the business environment because corruption has a negative influence on CSR performance. Organizations should develop infrastructure based on ethical corporate culture, a long-term business model, and a strong compliance framework. Therefore, it may be assumed that excellent CSR performance encourages businesses in combating risk-engaging in corrupt activities (Reyes-Menendez et al., 2018).

### E-Government Development and Corporate Social Responsibility Performance

The different economies and global agencies are becoming more concerned about their CSR practices. In this scenario e-government is an endeavor to utilize and use telecommunications to enhance government effectiveness and efficiency, deliver better service to the community, provide more accessibility, and make government more responsible and transparent to society (Elbahnasawy, 2021). E-government plays a significant part in anti-corruption policies and helps organizations to enhance their CSR performance (Suhendi et al., 2020). Several prior research has illustrated e-government development has a beneficial influence on CSR performance (Azad and Faraj, 2014; Khan and Krishnan, 2019; Arayankalam et al., 2021). Based on the above-mentioned literature, we presented our hypothesis as follows:

*H1: E-government has a positive impact on CSR performance.*

### E-Government Development and Mandatory Corporate Social Responsibility Policy

E-government allows the government to provide services to citizens in a more convenient manner. An e-government framework must restrict the strategic policies and phases of e-government in terms of their execution and development. With CSR activities, firms can gain multiple benefits such as long-term self-interest, greater stakeholder’s interest, and enhanced public image (Krishnan and Teo, 2012; Lee et al., 2016; Roblek et al., 2020). Several governments have lost patience with a firm’s willingness to make adequate voluntary contributions to CSR activities. Therefore, governments have taken steps to make mandatory CSR policy for promoting transparency and accountability. Gatto (2002) claims that mandatory CSR enhances corporate commitment and empowers numerous external stakeholders around firms. The use of mandatory CSR policy has been recommended as a way to persuade certain businesses to invest in CSR initiatives (Mukherjee et al., 2018). Based on the above-mentioned literature, we proposed our hypothesis as follows:

*H2: E-government has a positive impact on the mandatory CSR policy.*

### E-Government Development and Corruption

Corruption is termed as misuse of public office for personal benefits. Corruption may be caused by selling the government property, bribery, kickbacks in government services, and misappropriation of state funds (Lu et al., 2021). E-government development in the country has a protentional effect on reducing corruption in organizations (Hardy and Williams, 2008). Similarly, Kim et al. (2009) argued corruption may be reduced through the development of e-government, transparency, and strong and efficient leadership. The firms that deliberately operate in illegal and corrupt situations cause critical damage to an organization’s reputation (Ioannou and Serafeim, 2012; Keig et al., 2015). The level of corruption differs considerably among countries as a manifestation of the country’s legal, cultural, economic, and political system (Kong et al., 2021). Based on the above-mentioned literature, we proposed our hypothesis as follows:

*H3: E-government has a negative impact on corruption.*

### E-Government Development and Information and Communication Technologies Development

E-government is endeavored to create electronic services to improve the quality of services given to its stakeholders such as businesses, employees, residents, and other government entities. The ICTs development has resulted in the growth of e-participation, in which governments employ digital technologies to enhance stakeholders’ participation and e-government development. ICTs development is also found as a crucial component of a country that may serve as a foundation for e-government implementation. As ICTs development creates an effective mechanism for ICT access and structuring e-government (McKnight et al., 2002; Dilham et al., 2020). According to previous literature, a direct link exists between e-government and ICT development (Ojha and Palvia, 2012). Based on the above-mentioned literature, we presented our hypothesis as follows:

*H4: E-government has a positive impact on ICT development.*

### E-Government Development and Institutional Quality

E-government is a system that uses ICT as a tool to make communications and transactions among people, business organizations, and government agencies (Androniceanu et al., 2020; Roblek et al., 2020). ICTs in the e-government context may be a cost-effective and easy way of ensuring open and transparent government, which leads to reduced corruption in systems (Bertot et al., 2010). Countries that have achieved success in enacting transparency legislations have been linked to based efforts via e-government (Maqsoom et al., 2021b). ICTs promote good governance, boost reform-oriented efforts, improve connection among government personnel as well as monitor and manage government employee and project behavior (Chunhui Huo et al., 2020). As a result of e-government advancement, institutions can become more transparent in their managing information and procedures (Chang and Chu, 2006; Hwang and Choi, 2017; Dahwan and Raju, 2021). Based on the above discussions, we proposed our hypothesis as follows:

*H5: E-government has a positive impact on institutional quality.*

### Mandatory Corporate Social Responsibility Policy and Corporate Social Responsibility Performance

A strand of literature on corporate social responsibility has documented many incentives for the company to participate in CSR initiatives. The organizations with high CSR score are believed to be morally responsible and have minimum risk of corruption (Nawaz et al., 2019; Maqsoom et al., 2021a). CSR activities provide incentives to an organization in several ways such as CSR initiatives entail the better engagement of a corporation with its major stakeholders and increase the firm’s reputation. Moreover, participating in CSR activities can help to reduce the risk of unfavorable regulatory, legislative, and budgetary actions (Androniceanu et al., 2020). The use of mandatory CSR policy has been recommended as a way to persuade certain businesses to invest in CSR initiatives (Krishnan and Teo, 2012). The implications of mandatory CSR policy enable business leaders to better understand more clearly local stakeholders’ issues and manage their intermediaries (Khan et al., 2019). This shows that mandatory CSR policy significantly influences CSR performance.

*H6: Mandatory CSR has a positive impact on CSR performance.*

### Corruption and Corporate Social Responsibility Performance

Corruption is referred to as the misuse of economic and social power for personal objectives. Companies engaging in corrupt activities due to their corrupt operations lead to a negative effect on performance (Richey et al., 2005). Corruption at the organizational level is usually performed by the employees and managers on behalf of the corporation (Chedrawi et al., 2020). Managers and employees are more likely to participate in unlawful conduct when the company’s transparency and morale are low. Strategic and management flaws, as well as weak corporate ethics and misbehaviors, lead to ethical and social degradation. Firms must build strategic infrastructure that monitors and rectifies any unlawful activity to successfully prevent such risks particularly corruption (Idowu and Towler, 2004). Organizations should develop infrastructure based on ethical corporate culture, a long-term business model, and a strong compliance framework. Therefore, it is reasonable to assume that excellent CSR performance encourages businesses in combating risk-engaging in corrupt activities (Awan et al., 2021). Based on the above discussion, we proposed the hypothesis as follows:

*H7: Corruption harms CSR performance.*

### Information and Communication Technologies Development and Corporate Social Responsibility Performance

Numerous studies have described ICT development as it has a significant influence on CSR performance. ICT as a technical solution can significantly enhance the effectiveness of individuals and organizations (Tuan, 2018). Indeed, ICT has an influence on working habits and practices as well as relationships within an organization. The contribution of ICT to the company may also be viewed as the creation of specialized capability that allows the firm to gain a competitive edge over its competitors (Liang et al., 2010). The corporations that paid more attention to the development of CSR activities enjoy a good reputation among their stakeholders (Vahdati et al., 2015). Particularly, in this era of ICTs development, any information related to corporations like their opinions on CSR can influence stakeholders’ decisions (Chunhui Huo et al., 2020; Nawaz et al., 2020b). Based on the above-mentioned literature, we proposed our hypothesis as follows:

*H8: ICT development has a positive impact on CSR performance.*

### Institutional Quality and Corporate Social Responsibility Performance

The institutional quality of a country serves as an intermediary for efficient e-government development. ICTs in the e-government context may be a cost-effective and easy way of ensuring open and transparent government which leads to reduced corruption in systems (Tang et al., 2020). Countries that have success in enacting transparency legislation have been connected to based efforts via e-government (Carvalho et al., 2010). A strong country requires adequate financial market laws, a robust rule of law, intellectual property rights, and a high-quality institution capable of successfully combatting corruption (Hur and Kim, 2017). The ICTs development has resulted in the growth of e-participation and institutional quality in which governments employ digital technologies to enhance stakeholders’ participants and e-government development (Frankental, 2001).

*H9: Institutional quality has a positive impact on CSR performance.*

### Mediating Role of Mandatory Corporate Social Responsibility Policy

Mandatory CSR policy is usually policy implemented by the regulatory bodies and institutions on organizations to follow some set of obligations of CSR not for the sake of profit but imposed by law to follow (Moisescu, 2015). During the e-government initiatives, some developing countries recently imposed a mandatory CSR policy like India has mandated the mandatory CSR policy in the Indians Companies Act 2013 (Section-135) (Bhattacharyya and Rahman, 2019). The imposition of mandatory CSR policy in organizations does not only improve CSR performance but firm performance as well (Nawaz et al., 2021).

Moreover, today’s firms are not solely fulfilling the mandatory CSR requirements but also on other CSR practices to remove sustainability risk either social or economic (Hossan Chowdhury and Quaddus, 2021). Therefore, organizations are focusing on CSR practices to improve the overall CSR performance for the best interest of stakeholders and fulfilling the stakeholders’ expectations in CSR. Additionally, mandatory CSR improves the firm performance as well as has a strong positive impact on the externalities; moreover, areas with mandatory CSR policies helped to decrease the CO_2_ emission level and the wastewater in China (Chen et al., 2018). Therefore, we propose that,

*H_10_: Mandatory CSR policy mediates the relationship between E-government and CSR performance.*

### Mediating Role of Corruption

Corruption has been a terrible element in the development of countries. Government always tries to eliminate and minimize it but somehow it exists in every phase of government. But the e-government initiatives can minimize corruption in places like Nigeria (Adam, 2020). Corruption is significantly related to corporate social responsibility and cultural elements and corruption are key elements of corporate social responsibility (Agyei-Mensah and Buertey, 2019). Moreover, Agyei-Mensah and Buertey (2019) argued that corruption is a part of culture because it depends on how the people and society understand the rules and guidelines in a state or community. Moreover, the performance of corporate social responsibility is negatively linked with the risk of corruption therefore firm-level determinants are related to corruption risk (Lopatta et al., 2017). Another study by Chantziaras et al. (2020) investigated the association between religious norms and CSR reporting and found a weaker positive relationship between these factors, particularly where there is a higher corruption level. Based on previous studies, the author proposes that,

*H_11_: Corruption mediates the relationship between e-government and CSR performance.*

### Mediating Role of Information and Communication Technologies Development

Information and communication technologies development plays a crucial role in terms of introducing an e-government initiative because it provides a suitable platform for them to launch e-government initiatives to irradicate corruption in the country (Adam, 2020). ICT development has an important relationship with sustainability as ICT development creates enormous benefits for e-business and sustainable development (Nicolae and Sabina, 2012). E-government advancements may result in more public access to ICTs, as well as improved skills and training for using e-government networks. This means that a country’s e-government initiative might lead to a far broader ICT development agenda than when ICT advancements are undertaken to support specific government initiatives (Adam, 2020). Moreover, ICT development influences CSR engagement (Hendricx, 2020). There is limited evidence of ICT, e-government, and CSR; thus, we propose the hypothesis as,

*H12: ICT development mediates the relationship between e-government and CSR performance.*

### Mediating Role of Institutional Quality

The mediating role of institutional quality between e-government and corruption was studied by Adam (2020). He found that institutional quality is important to eliminate corruption in the country as well as provides a potentially strong platform to launch e-government initiatives. Interestingly, organizational related factors are also important as organizational size also leaves a significant impact on CSR engagement (Hendricx, 2020). Moreover, Heckelman and Powell (2010) suggested that institutional quality deteriorates, corruption severely damages growth, therefore confirming the claim that political institutional quality influences the connection between corruption and growth. Corruption is detrimental to economic progress, and it is exacerbated in nations with poor political systems (Méon and Sekkat, 2005). Evidence has lately emerged suggesting the rule of law may help to mitigate the link between transparency and corruption (Dar et al., 2021). Therefore, the author proposes that,

*H13: Intuitional development mediates the relationship between e-government and CSR performance.*

### Stakeholder’s Theory

**FIGURE 1:**
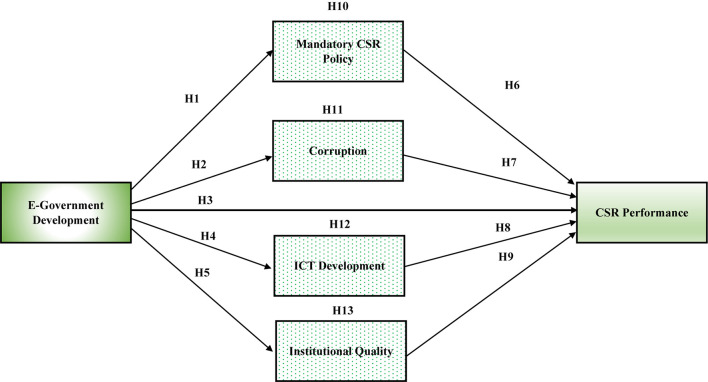
The conceptual model.

In this study, the stakeholder’s theory of CSR is used to explain the influence of e-government development on CSR performance through the mediating role of ICTs, CSR policy, corruption, and institutional quality. Freeman and Medoff (1985) defined stakeholders as “any individual or group who has an effect on or is influenced by the achievements of organizational goals.” All the stakeholders external and internal such as employees, consumers, governments, creditors, media, etc. may influence the performance of an organization (Nawaz et al., 2021; Qin et al., 2021). Corporations that perform well in terms of CSR activities may also be able to establish a strong bonding with their stakeholders (Cho et al., 2019). Therefore, when a company discloses its CSR, it is indicating that the company is socially responsible by communicating its attitude, activities, and outcomes in response to claims of its stakeholders. This study proposes 13 hypotheses and provides theoretical background. A graphical representation is presented in Figure 1.

## Research Methods

This study analyzes the relationship of e-government on CSR performance and the mediating effect of mandatory CSR policy on corruption and ICT development. The overall research philosophy is positivism. Positivism is where research is conducted to investigate the positive relationship between variables (Nawaz et al., 2020a). This is a deductive research by design where authors usually propose hypotheses and then test them. This quantitative study is based on a cross-sectional study where data is collected in single point time. To analyze the quantitative data, a structured questionnaire technique was used that covers 30 items. The data was collected through a convenience random sampling. A convenience random sampling technique is when the researcher collects data randomly from those respondents who are easily accessible to researchers (Nawaz et al., 2021). The data was collected from the managerial staff and top management stakeholders from China. Overall, 305 responses were collected to maintain the reliability of outcomes.

There are a few questions related to demographics that will be added to understand the description such as respondent’s demography which includes age, gender, education, current position, and experience. The data will be collected through a structured questionnaire and the data is collected from China so based on top management focused the questionnaire is made in English as many of the top managerial staff understand English as a language. To measure all of these variables, this study considered the instruments and scale from the previous studies.

### Instrument Development

All the constructs in the model will measure through the previous studies’ measurements. The e-government will be measured through three indicators (online service index, telecommunication infrastructure index, and human capital index), the ICT development through ICT development index based on three indicators (access, skill, and use), the institutional quality will be measured through Country Policy and Institutional Assessment (CPIA) index by World Bank based on four indicators (economic management, policies for social equity/inclusion, public sector management, and institutions and structural policies). We developed this measurement scale of all these constructs based on the previous indicators and analyzed the reliability and validity analysis through CFA and EFA or PLS-algorithm in Smart-PLS. We proposed a five-point Likert scale that varies from strongly disagree (1) to strongly agree (5). The questionnaire is made on the previous indicators used in the study (Adam, 2020). For further constructs, the Mandatory CSR Policy construct will be measured through four indicators introduced by the author based on previous studies (Cheng and Kung, 2016; Ramdhony, 2018). Moreover, for corruption, four indicator scales will be modified from the study of Mahmoudi and Bagheri Majd (2021). In the end, the CSR performance will be measured from Liu et al. (2014) study based on 10 items.

## Data Analysis

The data analysis is mainly based on three stages: demographic and descriptive statistics, measurement model assessment, and structural model assessment. The suggested conceptual model was tested using the Smart-PLS version 3.3.3 software, which employed the Smart-Partial Least Square Structural Equation Modeling (PLS-SEM). The method is divided into two parts: measurement model evaluation and structural model evaluation. According to a previous study, these two processes should be turned off simultaneously. The measurement evaluation exposes how the model’s variables are measured, whereas the structural model evaluation reveals how the model’s variables are linked.

The demographic details are illustrated in Table 1. Demographic summary delineates those respondents that are both male and female with the percentage of 52.79 and 47.21%, respectively. Respondents belong to multiple age groups (<25, 25–30, 31–40, 41–50, 50>). Respondents share multiple education qualification levels; however, around 70% of them have bachelor’s and master’s degrees. These respondents were from four different industries which are more related to the nature of the study such as information technology, online services, online education, and logistics and supply chain. This information demonstrates the profile of the focus group in this study and the diversity of information will help to generalize these findings.

**TABLE 1:** Demographic summary.

Summary	Frequency	%
***Gender***		
Male	161	52.79
Female	144	47.21
***Age***		
<25	120	39.34
25–30	89	29.18
31–40	45	14.75
41–50	30	9.84
50>	21	6.89
***Education***		
Higher secondary	39	12.79
Bachelor	134	43.93
Masters	80	26.23
Doctorate	24	7.87
Others	28	9.18
***Industry type***		
Information technology	121	39.67
Online services	80	26.23
Online education	45	14.75
Logistics and supply chain	59	19.34

*n = 305.*

**FIGURE 2:**
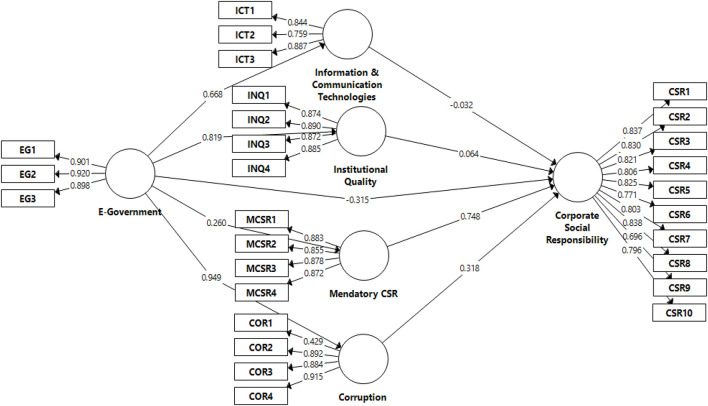
Partial least square-algorithm for the measurement model.

The second stage of analysis is measurement model assessment that is usually the first stage in the sequential equation modeling (SEM) in which the reliability validity is being measured for all the constructs in the conceptual model and indicators of these constructs. Further, in the measurement model, there are two validity measures overall: discriminant and convergent validity and reliability measures such as CR and Cronbach Alpha. The results of the measurement model are illustrated in Table 2 and a graphical representation is in Figure 2.

**TABLE 2:** Measurement model and descriptive statistics.

Constructs	Code	FD	α	CR	AVE	M	SD
e-government			0.891	0.932	0.821	3.895	1.035
	EG1	0.901					
	EG2	0.92					
	EG3	0.898					
Information and communication technologies			0.775	0.87	0.692	3.948	1.054
	ICT1	0.844					
	ICT2	0.759					
	ICT3	0.887					
Institutional quality			0.903	0.932	0.775	3.936	1.038
	INQ1	0.874					
	INQ2	0.89					
	INQ3	0.872					
	INQ4	0.885					
Mandatory CSR			0.895	0.927	0.761	3.888	1.101
	MCSR1	0.883					
	MCSR2	0.856					
	MCSR3	0.878					
	MCSR4	0.872					
Corruption			0.795	0.874	0.65	3.872	1.044
	COR1	0.43					
	COR2	0.892					
	COR3	0.884					
	COR4	0.914					
Social corporate responsibility			0.931	0.942	0.645	3.795	1.033
	CSR1	0.834					
	CSR2	0.826					
	CSR3	0.815					
	CSR4	0.810					
	CSR5	0.825					
	CSR6	0.777					
	CSR7	0.805					
	CSR8	0.847					
	CSR9	0.704					
	CSR10	0.805					

*FD, factor loadings; CR, construct reliability; AVE, average variance extracted; α, Cronbach Alpha.*

Convergent validity measures the extent to which the measures are correlated with each other. Constructs reliability is measured through CR and Cronbach Alpha as the values of both CR and Cronbach Alpha are greater than the threshold point which is 0.70 (Hair et al., 2017; Haq and Awan, 2020; Haq et al., 2020). Thus, the reliability of constructs is maintained, and all constructs are reliable to use for further analysis. Convergent validity is measured through factor loadings and AVE values. The thresholds for AVE and factor loadings are 0.50 and 0.70, respectively (Hair et al., 2017; Haq et al., 2020). All values for AVE are above 0.50 and factor loadings are greater than 0.70; therefore, the convergent validity is maintained and satisfactory.

Discriminant validity was measured through the Fornell and Larcker ratio criterion and Heterotrait-Monotrait (HTMT) ratio of association or correlation. The Fornell and Larcker ratio criterion is a measure of discriminant validity where all diagonal values in the table must be larger than their beneath values (Hair et al., 2017; Haq and Awan, 2020).

**FIGURE 3:**
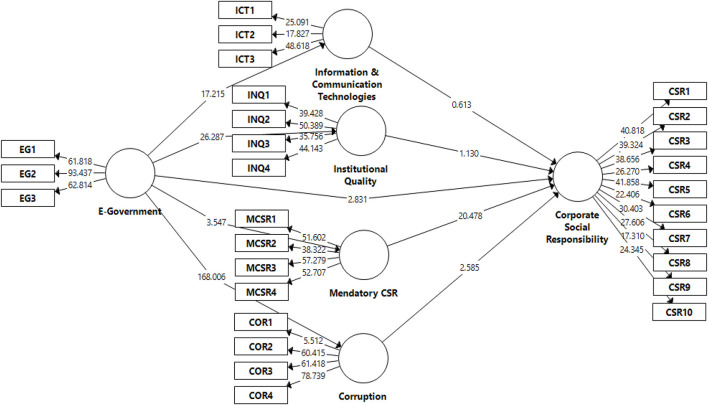
Partial least square-bootstrapping for the structural model.

Table 3 illustrates that all values in bold on the diagonal are greater than their below values. Thus, the condition of discriminant validity was met. Moreover, the second measure to estimate discriminant validity is the HTMT ratio where all values must be less than 0.90 (Hair et al., 2017). Thus, all values are less than the threshold point other than a few values as illustrated in Table 4. There is a bit of multicollinearity problem found in HTMT; however, the results of the Fornell and Larcker ratio criterion are appropriate. The structural model assessment is the third stage in analysis but that is usually the second stage in SEM. The structural model estimates the path or relationships between variables. It includes both direct and indirect effects. Direct effects are defined as the relationship between two variables; however, the indirect effects are mediating the roles of other constructs. This study employed *t*-statistics, *p*-values, *R*^2^, and Beta values or (O) to confirm the statistical significance and the direction either positive or negative and results for measurement and structural model assessment are illustrated in Table 5 and Figure 3. The H1 is tested and confirmed as there is a negative and significant association between e-government and CSR with *t*−*statistic* = 2.938:*p* − *value* = 0.002. Likewise, the next four hypotheses were also accepted and predicted a positive significant relationship of e-government with mandatory CSR, corruption, ICTs development and institutional quality with *t* − *statistic* = 3.436:*p* − *value* = 0.000, *t* − *statistic* = 167.619:*p* − *value* = 0.000, *t* − *statistic* = 17.319: *p* − *value* = 0.000, and *t* − *statistic* = 26.042: *p* − *value* = 0.000, respectively. Moreover, among the next four hypotheses such as (H6, H7, H8, and H9), H6, H7, and H9 were accepted with *t* − *statistic* = 20.082: *p* − *value* = 0.000, *t* − *statistic* = 2.657: *p* − *value* = 0.004, and *t* − *statistic* = 1.439: *p* − *value* = 0.075, however, the H8 were rejected where *p*-value is not satisfactory thus no statistical significance was found between ICTs development and CSR performance *t* − *statistic* = 0.696:*p* − *value* = 0.243.

**TABLE 3:** Fornell and Larcker criterion.

	CSR	COR	EG	ICTs	INQ	MCSR
**CSR**	**0.803**					
**COR**	0.343	**0.806**				
**EG**	0.218	0.949	**0.906**			
**ICTs**	0.063	0.649	0.668	**0.832**		
**INQ**	0.291	0.834	0.819	0.633	**0.880**	
**MCSR**	0.804	0.381	0.260	0.074	0.307	**0.872**

*CSR, corporate social responsibility; COR, corruption; EG, e-government; ICTs, information and communication technologies; INQ, institutional quality; MCSR, mandatory corporate social responsibility. The meaning of the bold values is shows significance.*

**TABLE 4:** HTMT ratio.

	CSR	COR	EG	ICTs	INQ	MCSR
**CSR**	**–**					
**COR**	0.492	**–**				
**EG**	0.243	1.088	**–**			
**ICTs**	0.105	0.788	0.802	**–**		
**INQ**	0.319	0.971	0.913	0.752	**–**	
**MCSR**	0.873	0.547	0.291	0.152	0.341	**–**

*CSR, corporate social responsibility; COR, corruption; EG, e-government; ICTs, information and communication technologies; INQ, institutional quality; MCSR, mandatory corporate social responsibility.*

**TABLE 5:** Direct and indirect effects.

Paths	H	(O)	(M)	(STDEV)	*T* Statistics	*P* Values	*R* ^2^	Results
EG **→** CSR	*H1*	–0.322	–0.314	0.109	2.938***	0.002	0.66	Supported
EG **→** MCSR	*H2*	0.26	0.258	0.076	3.436***	0.000	0.07	Supported
EG **→** COR	*H3*	0.949	0.949	0.006	167.619***	0.000	0.90	Supported
EG **→** ICTs	*H4*	0.668	0.672	0.039	17.319***	0.000	0.45	Supported
EG **→** INQ	*H5*	0.819	0.819	0.031	26.042***	0.000	0.67	Supported
MCSR **→** CSR	*H6*	0.743	0.745	0.037	20.082***	0.000		Supported
COR **→** CSR	*H7*	0.321	0.315	0.121	2.657***	0.004		Supported
ICTs **→** CSR	*H8*	–0.037	–0.039	0.054	0.696	0.243		Not supported
INQ **→** CSR	*H9*	0.082	0.083	0.057	1.439**	0.075		Supported
EG **→** COR **→** CSR	*H10*	0.305	0.298	0.114	2.666***	0.004		Supported
EG **→** MCSR **→** CSR	*H11*	0.193	0.192	0.056	3.472***	0.000		Supported
EG **→** ICTs **→** CSR	*H12*	–0.025	–0.026	0.036	0.691	0.245		Supported
EG **→** INQ **→** CSR	*H13*	0.067	0.068	0.047	1.431**	0.076		Supported

*** = 0.005%, ** = 0.10% significance level. H, hypothesis; O, original sample; M, sample mean; STDEV, standard deviation; CSR, corporate social responsibility; COR, corruption; EG, e-government; ICTs, information and communication technologies; INQ, institutional quality; MCSR, mandatory corporate social responsibility.

Four indirect effects (mediating variables) were introduced and proposed. Among those four mediating roles, three of them were accepted. The hypothesis H10 confirmed that corruption positively mediated the relationship between e-government and CSR where it implies that it is a full mediation with *t* − *statistic* = 2.666: *p* − *value* = 0.004. Likewise, H11 and H13 confirmed and mandatory CSR and institutional quality proved a positive mediation between e-government and CSR. These mediating effects were also proved as full mediation under *t* − *statistic* = 3.472: *p* − *value* = 0.004 and *t−statistic* = 1.431: *p* − *value* = 0.004. But the H12 were rejected where *t* − *statistic* = 0.691: *p* − *value* = 0.245.

## Discussion and Implication

Social corporate responsibility is an important factor for business success these days. Besides, it offers a productive workplace environment. Moreover, social corporate responsibility got great attention from government participants and policymakers in current circumstances due to Covid-19. This study explores the relationship between e-government and corporate social responsibility. Moreover, this study explores the direct and indirect effects. In other words, this study explores the mediating role of corruption, mandatory CSR, institutional quality, and ICTs development. This study is the first in the previous strand that investigated the direct effect of e-government and social corporate responsibility. Covid-19 forced a rapid e-government focus and electronic development and CSR is a core aspect of the recovery plan from the Covid-19 crisis (Bae et al., 2021). However, the first hypothesis showed that due to Covid-19 e-government is negatively associated with CSR these days. The direct hypothesis proved a positive impact of e-government on corruption, mandatory CSR, and institutional quality. The reason behind increased corruption is the lack of Internet access and information asymmetry. In contrast, mandatory CSR policy, corruption, and institutional quality were found to be positively linked with CSR. Moreover, the efficiency of e-government development is mediated by a country’s institutional quality (Adam, 2020). Likewise, the positive impact of corruption, mandatory CSR, and institutional quality on CSR implies that these factors are crucial for CSR performance and play a vital role in increasing the CSR overall in Covid-19. Some developing nations have lately implemented an obligatory CSR policy as part of their e-government ambitions, such as India, which has legislated a mandatory CSR policy under the Indian Companies Act 2013 (Section-135). The implementation of a mandated CSR policy in businesses improves not just CSR but also company performance (Bhattacharyya and Rahman, 2019). Furthermore, today’s businesses are focusing on various CSR activities in addition to the statutory CSR obligations to mitigate social and economic sustainability risks (Hossan Chowdhury and Quaddus, 2021). This study is based on the effect of e-government on CSR performance. The findings of the research will provide policy implications for those countries which introduce initiatives of e-government. This will also provide implications for the policymakers to introduce e-government programs for better CSR performance in the country. Additionally, this study will bring insight for the regulatory authorities to focus on improving institutional quality and ICT development. The development of ICT provides support to launch effective and successful e-government initiatives for governments. Moreover, the relationship of corruption with CSR performance will produce significant implications for corruption control measures and adverse effects of corruption on CSR performance. It will also give a picture of how mandatory CSR policy can be useful in terms of improving the overall CSR performance of the country or state. This means that solid institutional foundations and a robust legal system may serve as strong pillars for the growth of e-government. It is critical to building institutions so that citizens do not seek consolation in the pre-e-government environment’s physical bureaucracy, which fostered corruption.

## Conclusion

Nowadays, social corporate responsibility is a critical component of company success. It also provides a productive work atmosphere. Furthermore, due to Covid-19, social business responsibility has received a lot of attention from government players and politicians. Therefore, the link between e-government and corporate social responsibility is investigated in this study. PLS-SEM research analysis technique was employed using Smart-PLS 3.3.3 as an analysis tool. This study is based on survey and structured questionnaire. Moreover, the data was collected through convenience sampling. The findings of the study reveals that e-government has a negative impact on CSR performance. The e-government meaningfully predicts the corruption, ICTs development, mandatory CSR discourse, and institutional quality. However, these constructs are positively related with CSR performance. Mandatory CSR, institutional quality, and corruption proved to be a mediator between e-government and CSR performance. This study has several contributions to the current strand of literature. At first, this study aims to develop and investigate the relationship between e-government and corporate social responsibility during Covid-19. Second, this research investigated mandatory corporate social responsibility, institutional quality, ICTs, and corruption as mediators. This study aims to answer the question of how e-government impacts corporate social responsibility and how this relationship can be influenced by other mediating forces. Therefore, government and related stakeholders should consider these findings to form the policy related to e-government. There are a few limitations to this study. The data for this study was obtained at a single moment in time and is cross-sectional. As a result, longitudinal research can give a more accurate representation of current results. Second, this research took into account the opinions of respondents and was based on primary data. As a result, it accurately depicts what Chinese citizens and experts believe. Secondary data and actual data, on the other hand, can provide a more realistic picture of China’s administration and its ramifications. Finally, because this research was done in China, the results cannot be applied to other nations. As a result, these findings may not apply to nations that are not focused on e-government. Future research should explore the related concepts, i.e., e-banking service quality (Haq and Awan, 2020) with CSR.

## Data Availability Statement

The original contributions presented in the study are included in the article/supplementary material, further inquiries can be directed to the corresponding author.

## Author Contributions

AARNA conceived and designed the concept literature review, data collection, and wrote the manuscript. YC, AA, and YY helped to provide technical support and contributed to analysis tools. AARNA and TS reviewed the work to improve the outcomes. All authors read and agreed to the published version of the manuscript.

## Conflict of Interest

The authors declare that the research was conducted in the absence of any commercial or financial relationships that could be construed as a potential conflict of interest.

## Publisher’s Note

All claims expressed in this article are solely those of the authors and do not necessarily represent those of their affiliated organizations, or those of the publisher, the editors and the reviewers. Any product that may be evaluated in this article, or claim that may be made by its manufacturer, is not guaranteed or endorsed by the publisher.
